# Omics-based characterization of physiological and transcriptomic resilience during cold de-acclimation and re-acclimation in GSK-like kinase mutants

**DOI:** 10.1186/s12870-026-09587-3

**Published:** 2026-07-22

**Authors:** Sunny Ahmar, Marcin Rapacz, Tahir Mahmood, Iwona Sadura-Berg, Anna Maksymowicz, Ewa Pociecha

**Affiliations:** 1 Department of Plant Breeding, Physiology and Seed Science, Faculty of Agriculture and Economics, University of Agriculture in Krakow, Podłużna 3, Krakow, 30-239 Poland; 2 State Key Laboratory of Cotton Bio-Breeding and Integrated Utilization, Institute of Cotton Research, Chinese Academy of Agricultural Sciences, Anyang, 455000 China; 3 The Franciszek Górski Institute of Plant Physiology, Polish Academy of Sciences, Niezapominajek 21, Krakow, 30-239 Poland

**Keywords:** Cold acclimation, Stress memory, Brassinosteroid signalling, Transcriptomics, Abiotic stress resilience, De-acclimation

## Abstract

**Background:**

Cold acclimation, de-acclimation, and re-acclimation are critical adaptive phases that determine plant survival under fluctuating temperatures. Although the physiological and molecular basis of initial cold acclimation is well studied, the regulatory mechanisms governing the loss and re-acquisition of freezing tolerance remain poorly understood. Brassinosteroid (BR) signalling, mediated by glycogen synthase kinase-like (GSK-like) kinases, modulates both stress tolerance and plant growth; however, its role under fluctuating cold-related temperature conditions has not been explored in detail. This study investigated the physiological and transcriptomic responses of *Arabidopsis thaliana* wild-type (Col-4) and two BR-signalling mutants carrying T-DNA insertions in GSK-like kinase genes BIL1, (*At2g30980)*; GSK3 (*At1g06390*) across cold acclimation, de-acclimation and re-acclimation phases.

**Results:**

All genotypes exhibited reduced leaf water content (~ 55%) and suppressed photosystem II efficiency during cold acclimation, with partial recovery during de-acclimation and re-acclimation. Notably, the genotypes showed divergent roles in re-acclimation responses: Col-4 showed the greatest freezing tolerance (LT₅₀ = -20 °C), whereas BIL1 and GSK3 mutants showed more moderate re-hardening (~-15 °C and − 13 °C, respectively). In contrast, both mutants showed higher post-freeze canopy regrowth (headroom recovery ~ 80%) compared with Col-4 (~ 20%) during de-acclimation, revealing a trade-off between maximal freezing tolerance and vegetative recovery. Chlorophyll fluorescence analysis showed that GSK3 achieved the strongest photosynthetic recovery, while BIL1 displayed persistently impaired electron transport efficiency. Transcriptomic profiling revealed a genotype-specific reprogramming switch: during de-acclimation, GSK3 produced the largest transcriptional response (1,433 differentially expressed genes), whereas during re-acclimation this pattern reversed, with BIL1 exhibiting the most extensive but poorly coordinated reprogramming (1,876 differentially expressed genes). The co-expression network analysis revealed a dense, highly connected re-acclimation module in GSK3, contrasting with a fragmented network architecture in BIL1, which indicates qualitatively different transcriptional coordination capacities between the two mutants.

**Conclusions:**

Our findings suggest that GSK-like kinase signalling may contribute to cold resilience, potentially influencing the balance between freezing tolerance, photosynthetic recovery, and transcriptional coordination under fluctuating cold temperature conditions. The BIL1 mutant enhances post-freeze regrowth but compromises re-hardening and coherent transcriptional reprogramming, whereas GSK3 supports stronger photosynthetic and transcriptomic recovery. These findings identify GSK-like kinases as candidate regulators that warrant further functional validation as potential targets for engineering cold resilience in crops exposed to increasingly variable thermal environments.

**Supplementary Information:**

The online version contains supplementary material available at https://doi.org/10.1186/s12870-026-09587-3.

## Introduction

Cold stress presents a significant threat to agriculture by directly restricting plant growth and yield, particularly in Eastern Europe as well as temperate and high-latitude regions. In response to cold regimes, plants have evolved a dynamic acclimation process known as cold acclimation (CA), which enhances freezing tolerance and enables survival under low, non‑freezing temperatures [1, 2]. This process involves significant remodelling at physiological, biochemical, and transcriptional levels, including osmotic adjustment, membrane stabilization, and reorganization of the photosynthetic machinery [3, 4]. However, prolonged periods of stable cold are rare in nature, and plants are frequently exposed to fluctuating temperatures that trigger repeated cycles of warming‑induced de‑acclimation (DA) followed by second cold exposure. Such frequent cold exposure often results in a partial or complete loss of acquired freezing tolerance, thereby increasing plant vulnerability to subsequent freezing events [5–7]. The mechanisms underlying CA have been extensively investigated in various model plants and crop species. However, the regulatory processes governing DA and RA are not well understood, especially regarding their influence on plant physiological and transcriptomic responses. DA is not merely the reverse of acclimation; rather, it represents an active phase characterized by metabolic reactivation, hormonal homeostasis, and transcriptional changes that promote plant growth and development processes [8]. In contrast, RA often involves the reactivation of stress-protective pathways in plants whose normal physiological functions remain partially inhibited [6, 8]. Several studies have indicated that these transitions are governed by complex regulatory networks rather than by a simple reactivation of cold-responsive pathways. These findings underscore the significance of stress memory and recovery dynamics [9–11].

Frequent exposure to low temperatures induces significant alterations in plant water relations and leads to a decline in photosynthetic efficiency. These effects are primarily attributed to the destabilization of cellular membranes, restricted diffusion of CO₂, and suppression of photosystem II activity [12]. Furthermore, extracellular ice formation causes cellular dehydration, resulting in significant reduction of relative water content (RWC) within the plant tissue. This water deficit initiates osmotic adjustment mechanisms, primarily through the accumulation of compatible solutes, which help to maintain cellular homeostasis and mitigate the detrimental effects of dehydration [7]. Under cold exposure, these dehydration‑driven changes increase dissipation of excess excitation energy and lead to a subsequent decline in electron transport efficiency within the photosynthetic activity [1, 13, 14]. Despite significant progress in elucidating the mechanisms of cold acclimation, the extent to which physiological responses are reorganized during successive cycles of cold exposure remains poorly understood. In addition, only a limited number of genotypes have been evaluated under repeated cold-exposure regimes, even among well-studied model species such as *Arabidopsis thaliana* and major crop plants. At the molecular level, plant responses to cold exposure are regulated through the *ICE-CBF-COR* transcriptional cascade, which coordinates the activation of a wide array of cold-regulated genes associated with osmoprotection, antioxidant defense, and stabilization of cellular membranes [15]. Beyond this central pathway, hormonal signalling networks that regulate both growth and stress responses, especially those involving abscisic acid (ABA) and brassinosteroids (BRs), have been identified as important regulators of plant adaptation to cold conditions [16]. BR signalling, mediated by glycogen synthase kinase-like (GSK-like) kinases such as *BIN2* and related proteins, modulates both stress tolerance and developmental processes through phosphorylation-dependent regulation of key transcription factors [17]. For example, *BIN2* interacts with and phosphorylates *INDUCER OF CBF EXPRESSION 1 (*ICE1), targeting it for proteasome-mediated degradation and thereby negatively regulating freezing tolerance during prolonged cold exposure [18]. Despite these insights, the role of BR-associated kinases in coordinating physiological and transcriptional responses during de-acclimation (the loss of cold hardiness during warming) and re-acclimation (the re-acquisition of hardiness) remains largely unexplored [7].

Several studies indicate that these phases are not simply physiological or molecular opposites of the initial cold-acclimation process; rather, they involve distinct, tightly regulated transcriptional programs. During these transitional stages, tolerant genotypes often exhibit a form of transcriptional memory that enables them to maintain, or more rapidly re-establish, cold-responsive gene-expression networks upon secondary exposure [19]. In addition, phytohormone signalling, particularly through BRs, plays a central role in modulating these transitional phases. BR signalling helps balance growth resumption after de-acclimation, protects chloroplast function under fluctuating temperatures by adjusting the shift between efficient photosynthesis and photoinhibition, and regulates the expression of stress-protective compounds to optimize resource allocation [20]. Despite these structural and hormonal insights, how this complex transcriptional coordination translates into whole-plant physiological recovery under cyclic cold exposure remains unresolved, especially when comparing network architectures across genetically diverse cultivars. The transcriptomic dynamics that occur during de-acclimation and subsequent re-acclimation remain poorly understood.

In this study, the effects of temperature fluctuations, specifically cold acclimation (CA), de-acclimation (DA), and re-acclimation (RA) both physiologically and at transcriptomic level in *Arabidopsis thaliana* wild type (Col-4) and two BR-signalling mutants (BIL1 and GSK3) were evaluated. We hypothesized that defects in BR-associated kinase signalling would differentially influence processes such as water relations and osmotic adjustment, photosynthetic energy partitioning, and transcriptional coordination during cold-related temperature fluctuations. By integrating physiological measurements, chlorophyll fluorescence analysis, differential gene expression profiling, and co-expression network analysis, this study aimed to elucidate the mechanisms underlying genotype-specific trade-offs between freezing tolerance, recovery capacity, and transcriptional organization. These findings provide new insights into how plants balance growth and stress responses under fluctuating thermal environments and highlight potential targets for improving cold resilience in crops.

## Materials and Methods

### Plant materials

The present study was conducted on *Arabidopsis thaliana* as a model plant species. Three genotypes were evaluated both physiologically and at the transcriptomic level in response to cold acclimation, de‑acclimation, and re‑acclimation processes (Figure S1). The wild‑type *Arabidopsis thaliana* accession Col‑4 (Columbia, N933) was used as the control, alongside two brassinosteroid (BR)‑signaling mutants, BIL1 (accession ID; N2996) and GSK3 (accession ID; N1015053). These mutants carry T‑DNA insertions within *At2g30980* and *At1g06390* gene, respectively, both of which encode members of the glycogen‑synthase‑kinase‑like (GSK‑like) kinase family involved in BR signal transduction pathways. The BIL1 (*At2g30980*) mutant line used in this study carries a T‑DNA insertion located at ATG + 1135. Plants homozygous for this insertion displayed no observable morphological differences from the wild‑type. The GSK3 mutant line carries an annotated T‑DNA insertion and was propagated through four successive generations prior to the experiments. During this period, no segregation was observed. This was readily apparent because the GSK3 mutant exhibited phenotype characterized by larger plant size compared with the Col-4 wild type. Seeds for all three cultivars were obtained from the European Arabidopsis Stock Centre (NASC), which distributes germplasm in coordination with the Arabidopsis Biological Resource Center (ABRC) at The Ohio State University, United States.

### Experiment design

The experiment was conducted under controlled conditions in a growth chamber using a randomized design consisting of three treatments (cold-acclimated, de-acclimated and re-acclimated). Seeds of each genotype were sown into multi-cell trays (50 × 30 cm) containing 60 cells. In each cell, a few seeds were initially sown; subsequently, seedlings were thinned so that three to four healthy plants per cell were retained for exposure to the experimental treatments. Afterwards, the multi-cell trays were maintained for three weeks under controlled conditions at 18 /18°C (day/night) with a photoperiod of 13 h/11 h (day/night) and 250 µmol m⁻² s⁻¹ PPFD (photosynthetic photon flux density). Illumination was provided by Philips SON‑T AGRO 400 W high‑pressure sodium lamps. Next, plants were exposed for a one-day pre-acclimation (PA) period, followed by cold acclimation (CA) at 4/2°C (day/night) under an 8/16 h photoperiod and a 200 µmol m⁻² s⁻¹ PPFD for 10 days. After the acclimation phase, the plants were de‑acclimated (DA) at 12/6°C (day/night) under the same photoperiod and light intensity for four days. Finally, the plants were re‑acclimated (RA) at 4/2°C (day/night) under a constant PPFD of 200 µmol m⁻² s⁻¹ for an additional four days.

### Turgidity measurements

The fully expanded mature leaf from the rosette was excised, leaving a petiole, after the last day of each treatment. Immediately after collection, the leaves were placed in a pre-weighed zipper-lock bag, and the total weight was recorded to determine their fresh weight. After determining the fresh weight, the leaves were immersed in distilled water inside a closed container for 24 h under dark conditions to obtain the full turgor weight (FTW). After reaching full turgor, the leaves were removed from the water, gently blotted to remove excess surface moisture, and weighed immediately to obtain the FTW. Dry weight (DW) was measured after drying the leaves for 24 h in a paper envelope in an oven at 60–80 °C until a constant mass was reached. Each measurement was taken in ten replicates. Relative turgidity was calculated using the formula:

$$\:RT=\frac{FW-DW}{FTW-DW}\times\:100$$

Where FW is fresh weight, FTW is full turgor weight, and DW is dry weight.

### Osmotic potential measurements

Osmotic potential in leaf tissues was evaluated using an HR 33T Dewpoint Microvoltmeter (Wescor, Inc., USA) equipped with a C‑52 sample chamber. The leaf sample was collected from the youngest fully expanded leaf during each treatment and immediately frozen in liquid nitrogen. The cell sap was squeezed from the leaves using a syringe with a constant force, then applied onto a filter paper disc and placed in the C52 sample chamber. Measurements were taken at the dew point after a 20-minute equilibration period. The instrument’s output voltage is directly proportional to the water potential, using a calibration factor of − 0.75 µV per bar (1 bar = 10⁵ Pa). Osmotic potential in the chamber was calculated from the thermocouple temperature coefficient using the equation $\:{\pi\:}_{v1}=0.7({T}_{1}-{T}_{0})+{\pi\:}_{v0}$. Each sample was measured eight times for every treatment and cultivar.

### Assessment of frost tolerance (LT_50_, percentage of survival, recovery after freezing)

Lethal temperature (LT₅₀), the temperature at which 50% of the plants are damaged, was determined to evaluate the cold hardiness of each cultivar in response to cold acclimation, de‑acclimation, and re‑acclimation. Estimation of LT_50_ was performed on whole leaves on the basis of electrolyte leakage tests following the method described by Rapacz (1999) [21]. The fully expanded youngest leaves were placed on ice in plastic vessels. These vessels were then cooled at a controlled rate of 0.2 K min⁻¹ until reaching the desired test temperatures (–5, − 9, − 15, and − 19 °C), where they were held for 1 h to induce controlled freezing injury. Afterwards, thawing was conducted for 24 h at + 2 °C on a laboratory shaker. The index of injury, following the method of Flint et al. [22], was calculated for each freezing temperature. LT₅₀ values were derived from regression analyses based on injury measurements obtained at several freezing temperatures. Because a single LT₅₀ estimate was generated for each treatment from the fitted injury–temperature relationship, no formal statistical comparison of LT₅₀ values was performed. In total, LT₅₀ measurements were performed with ten replicates at each freezing temperature for each treatment and for each cultivar.

The percentage of plants that survived after exposure to frost was assessed after cold acclimation, de-acclimation and re-acclimation. The plants were placed in freezing chambers at 2 °C and then the temperature was gradually lowered by 3 °C/h until the required frost level was reached (–5, − 9, − 15, and − 19 °C) and held at this level for a period of 3 h. Subsequently, the temperature was increased to 2 °C at the rate of 3 °C/h. After that, the multi-cell trays with plants were transferred to a growth chamber and left for regrowth at 12 °C (8/16 h (day/night) photoperiod) and 150 µmol m^− 2^ s^− 1^ PPFD. After 2 weeks, plant survival was estimated based on the percentage of living plants.

Post-freezing recovery of *Arabidopsis thaliana* accessions (Col-4, BIL1, GSK3) was quantified from paired top-view images acquired 1 day (D1) and 14 days (D14) after freezing. For each experiment, images were aligned to a common geometry and were partitioned into a fixed 6-column grid corresponding to the pot layout (columns 1–2: Col; 3–4: BIL1; 5–6: GSK3). Within each pot region of interest, green canopy cover (green cover, %) was estimated by color-based segmentation using a normalized greenness index (Excess Green/ExG), and an identical thresholding procedure was applied to D1 and D14 images. Recovery was expressed as (i) the absolute change in green cover, Δcover (percentage points, pp) = cover (D14) – cover (D1), and (ii) a headroom-corrected recovery index, headroom (%) = (cover(D14) − cover(D1)) / (100 − cover(D1)) × 100, by which the limited growth potential at high initial cover was accounted for. Pots were assigned to freezing temperatures according to their row positions within each experiment, and all temperatures were included in the final analyses. To limit the influence of aberrant observations, outliers were removed using Tukey’s rule (values outside Q1 − 1.5×IQR or Q3 + 1.5×IQR) computed separately for each experiment × accession group, and observations flagged as outliers in either Δcover or headroom were excluded prior to statistical comparisons and visualization. All image processing, quantification, and statistical analyses were performed in Python (NumPy, pandas, and statsmodels), and figures were generated using Matplotlib.

### Chlorophyll fluorescence parameters

Chlorophyll a fluorescence was measured in the Col‑4, BIL1, and GSK3 genotypes in response to the PA, CA, DA, and RA conditions using a Plant Efficiency Analyzer (Handy‑PEA fluorimeter, Hansatech Instruments Ltd., Pentney, King’s Lynn, Norfolk, England) on the last day after each treatment. Leaves were dark‑adapted for 30 min, and measurements were taken from the middle region of fully expanded youngest leaves. In total, measurements were taken using ten replicates for each treatment within each genotype. Raw data from the chlorophyll fluorescence measurements were processed to extract structural and functional parameters of the photosynthetic apparatus using the standard JIP‑test equations describing PSII photochemistry and energy fluxes [23]. All parameters were normalized to the corresponding PA treatment and expressed as a percentage of the control, enabling genotype‑specific comparisons. The following parameters were selected: Fv/Fm, Fv/Fo, ψ₀, Vj, Mo, Tfm, Area, ABS/RC, TRo/RC, ET_0_/RC, DIo/RC, RC/ABS, RC/CSo, RC/CSm, PIABS, PItotal, REo/RC, and φRo to describe the functional domains of PSII photochemistry and energy partitioning for each genotype in relation to each treatment.

Multivariate analyses were conducted to assess the variability in the photosynthetic shifts of each genotype in response to the different treatments. Radar plots were generated to visualize the relative changes in physiological state of the plants in response to CA, DA, and RA. Energy-use efficiency was assessed using the ET_0_/DI_0_ ratio. To further elucidate the distinct grouping patterns and variance across genotypes and treatments. Principal Component Analysis (PCA) and correlation analyses were calculated and visualize using Agglomerative Hierarchical Clustering applied to the normalized parameter matrices.

### RNA sample collection and sequencing

Fresh leaves from each genotype (Col‑4, BIL1, and GSK3) were collected 24 h after each treatment (CA, DA, and RA). For each genotype, leaves from five different plants were combined and treated as one biological replicate, and two biological replicates were prepared per treatment. The samples were collected from the middle portion of fully expanded leaves, with a total fresh weight of 0.03–0.05 g and were immediately frozen in liquid nitrogen and stored at − 80 °C until RNA extraction. A total of 18 samples were collected, and total RNA was extracted using the RNeasy Plant Mini Kit (Qiagen, Hilden, Germany). The quantity and purity of the RNA were assessed using a UV–Vis Q5009 spectrophotometer (Quawell, San Jose, CA, USA), and RNA integrity was verified with a 2100 Bioanalyzer (Agilent Technologies, Santa Clara, CA, USA).

A total of 18 RNA samples were sent to weSEQ.IT (Rybnik, Poland) for sequencing. During library preparation, mRNA was isolated using oligo(dT)-attached magnetic beads, and cDNA libraries were constructed, converted into single‑stranded circular DNA (ssCir DNA), and sequenced in paired‑end 100 bp mode on the DNBSEQ platform (BGI, Cambridge, MA, USA) with a sequencing depth of 20 million paired reads (40 million reads total). The raw reads generated were used for further processing.

### Identification of differentially expressed genes (DEGs) and functional enrichment

The resulting raw RNA‑seq reads were first subjected to a quality check using with FastQC (Braham Bioinformatics, https://www.bioinformatics.babraham.ac.uk/projects/fastqc/), and then filtered using the Cutadapt (version 1.4.2; Martin, 2011) Python package, and Fastp [24] was used to remove adapter sequences and low‑quality reads. The resulting clean reads were then mapped to the *Arabidopsis thaliana* reference genome obtained from TAIR (Arabidopsis_thaliana.TAIR10.dna.toplevel) using HISAT2 version 2.0.4 [25]. The expression levels of genes and transcripts were quantified using StringTie [26] and reported as Fragments Per Kilobase per Million mapped reads (FPKM), following the method described by [27]. Differential expression analyses were performed to generate pairwise comparisons for each genotype in response to CA, DA, and RA treatments. Gene‑level changes were assessed using the R package DESeq2 [28]. DESeq2 was applied directly to the raw read counts, with library-size normalisation and dispersion estimation performed within the package. Genes were classified as differentially expressed when they met the thresholds of FDR < 0.05 and fold change (FC) ≥ 2.

Functional enrichment analyses were performed to evaluate the biological roles of the identified DEGs. Enrichment was assessed using a hypergeometric test with a significance threshold of *p* < 0.05, following the framework described by [29]. Gene Ontology (GO) annotation and enrichment were performed using the topGO R package v2.18.0 [30], and KEGG pathway enrichment was carried out with the clusterProfiler R package v3.10.1 [31]. These enrichment analyses enabled the identified DEGs from each pairwise combination to be interpreted in terms of enriched biological processes, molecular functions, cellular components, and metabolic pathways associated with the CA, DA, and RA responses, as well as their genotype‑specific behaviour.

### Weighted gene co-expression network (WGCNA) analysis

A gene co-expression network was constructed using WGCNA package in R [32]. A soft thresholding power was used to achieve a scale free-topology fit index (R^2^) of > 0.85. The network was built using Pearson correlation, and the Topological Overlap Matrix (TOM) was calculated with a maximum block size of 3000. A minimum module size of 20 genes and a strict merge cut height of 0.10 were applied to define co‑expression modules. The Partitioning Around Medoids (PAM) refinement step was enabled and constrained to follow the dendrogram structure to ensure accurate and biologically meaningful module splitting. To assess the relationship between co‑expression modules and experimental traits, module eigengenes (MEs) were calculated and correlated with each genotype (Col‑4, BIL1, and GSK3) under CA, DA, and RA stress conditions. The module–trait associations were visualized using a heatmap to highlight genotype‑specific patterns of co‑expression. Modules showing the strongest absolute correlations (|r|) (BIL1_CAvsRA_Purple and GSK3_CAvsRA_Lightyellow modules) were selected for hub‑gene identification, network construction, and functional enrichment analysis.

### Statistical analysis

All statistical analyses were performed using multifactor analysis of variance (ANOVA) in RStudio, version 3.6.0. The graphs were plotted with standard error bars for each data point, and post‑hoc comparisons were performed using Duncan’s multiple range test with a significance threshold of *p* ≤ 0.05. Heatmaps and hierarchical clustering were generated using TBtools‑II, providing a clear visualization of expression patterns and relationships among samples and genes.

## Results

### Physiological responses to cold stress in BR‑signalling mutants of *Arabidopsis* – water management

**Fig. 1:**
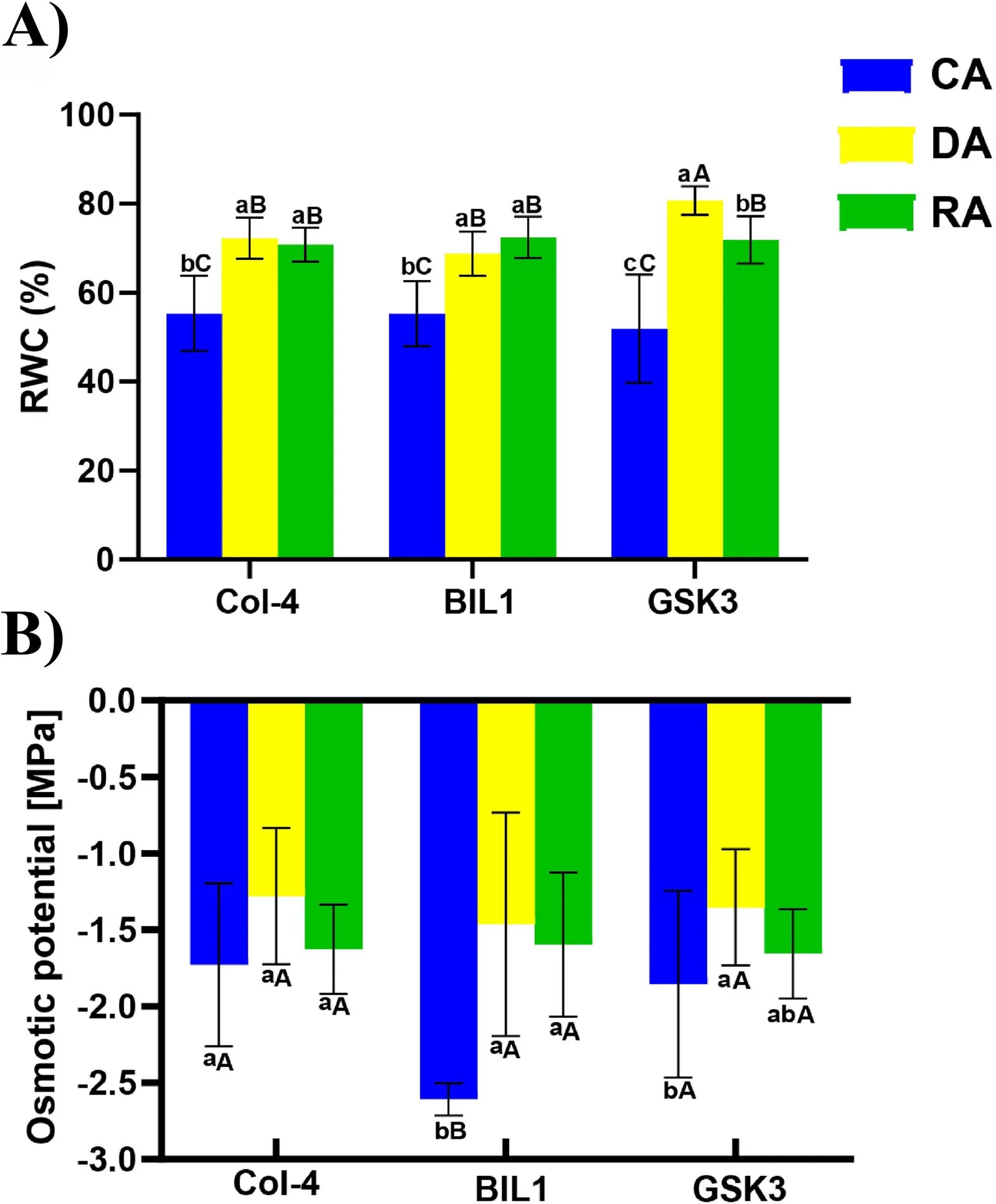
Physiological responses of Col-4, BIL1 and GSK3 during CA, DA and RA treatments. **A** Relative water contents (RWC): Capital letter shows significance level across genotype and treatment interaction across all samples; Small letter within each genotype: **B** Osmotic Potential measurement (OSM): Capital letter shows across genotype and treatment interaction across all samples; Small letter within each genotype

The wild type Col‑4 (Columbia) and the two BR‑signalling mutants (*AtBIL1* and *AtGSK3*) which carry T-DNA insertions in the GSK‑like kinase genes *At2g30980* and *At1g06390* were evaluated for their physiological responses during pre-acclimation (PA), cold acclimation (CA), de‑acclimation (DA), and re‑acclimation (RA) (Figure S1). The overall plant health and growth patterns of the wild type and the BIL1 and GSK3 mutants remained largely comparable, with similar canopy appearance and leaf morphology across the PA, CA, DA, and RA phases. To assess physiological changes during cold stress, we examined leaf water status across CA, DA and RA treatments. During CA, all three genotypes showed an initial reduction in tissue water content, with relative water content (RWC) decreasing to approximately ~ 55% (Fig. 1A). The GSK3 mutant showed a greater reduction than the Col-4 and BIL1. During DA, both the wild type and the mutants began to recover their water content, reaching up to 80%. Notably, the GSK3 mutant exhibited a significantly higher RWC recovery (~ 80%) compared with Col-4 and BIL1 (~ 70%). Furthermore, when plants were again exposed to a second cold exposure (RA), all genotypes showed relatively similar RWC values (~ 70%) which may reflect a more rapid re-adjustment water balance upon the second cold exposure.

Furthermore, leaf sap osmotic potential (OSM) highlighted a clear interaction between genotype and treatment, reflecting differences in solute accumulation that plants use as a defence mechanism during low temperature exposure (Fig. 1B). During CA, the BIL1 mutant showed significantly more negative osmotic potential (− 2.6 MPa) compared with Col-4 and GSK3, which showed values of approximately − 1.7 MPa and − 1.9 MPa, respectively. This stronger osmotic adjustment in BIL1 mutants suggests that the mutation in *At2g30980* enhances the plant’s internal defence capacity by promoting active solute accumulation during the first exposure to cold. However, during the DA and RA, the osmotic potential of both BIL1 and GSK3 mutants returned to values similar to those of the wild type (approximately − 1.2 to − 1.5 MPa), showing no statistically significant differences among the genotypes (Fig. 1B).

### Freezing tolerance and plant survival

**Fig. 2:**
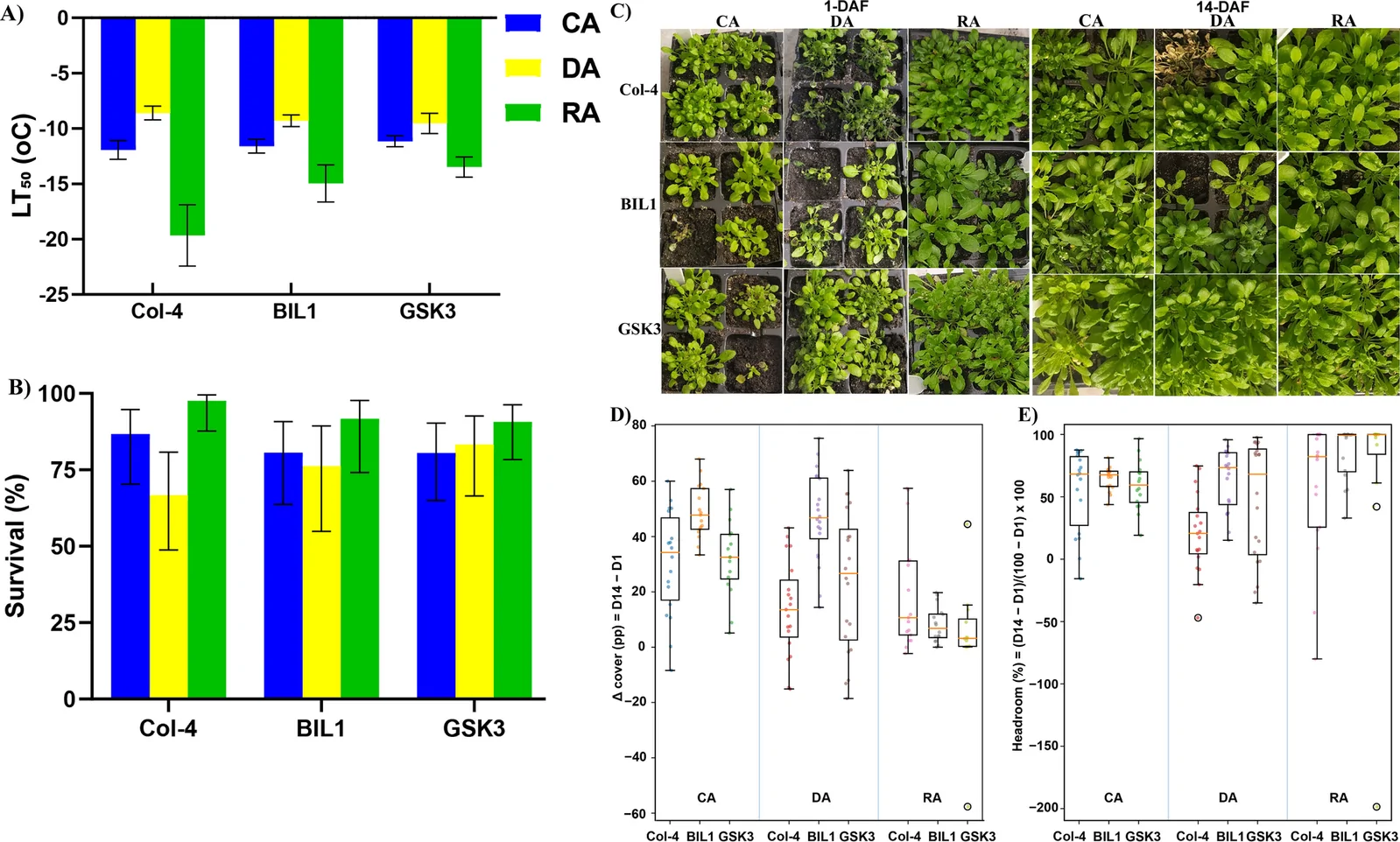
Freezing tolerance, post-freeze survival, and growth recovery rate of Arabidopsis genotypes under CA, DA and RA treatments. **A** LT_50_ values of Col-4, BIL1, and GSK3 in response to CA, DA, and RA. **B** Survival percentage of Col-4, BIL1, and GSK3 in response to CA, DA, and RA. Bars represent means± error bars. **C** Phenotypic visualization plant images at 1 and 14 days after freezing (1-DAF and 14-DAF), showing phenotypic responses of Col-4, BIL1, and GSK3 under CA, DA, and RA. **D** Change in rosette cover between 1 and 14 days after freezing, expressed as absolute difference in projected cover **E** Relative headroom or recovery index indicating the proportion of remaining growth capacity achieved after freezing. Boxplots show the distribution of individual plants, with points representing biological replicates

Cold hardiness of the three genotypes was assessed using freezing-tolerance measurements, including electrolyte leakage to determine LT₅₀, whole-plant survival and post-freezing canopy regrowth in response to CA, DA, and RA among genotypes. Lethal temperature (LT₅₀) showed significant differences among genotypes in their response during CA, DA and RA (Fig. 2A). In response to CA as initial exposure, all three-genotypes showed similar LT₅₀ values (~-11, -12 °C) as baseline of freezing tolerance, whereas, upon transfer to DA, the expected loss of cold hardiness was observed, with LT₅₀ values increasing to approximately − 8 °C to − 9 °C. In contrast, during the RA, the wild-type Col-4 exhibited a substantial increase in freezing tolerance, with LT₅₀ values dropping to nearly − 20 °C. The BIL1 and GSK3 mutants showed a more moderate frost tolerance, reaching LT₅₀ values of approximately − 15 °C and − 13 °C, respectively. These values indicate that functional GSK-like kinases are essential for plants to maximize their freezing tolerance during frequent or cold-related temperature fluctuations.

Post-freezing survival was also determined across all genotypes in response to CA, DA and RA. Overall, survival rates were high across all genotypes and treatments. However, genotypes showed slight differences depending on stress phase. In response to CA, survival rates were high across all genotypes (> 80%) indicating effective initial acclimation to cold stress. During DA, the survival rate of Col-4 declined to approximately 65%, whereas the BIL1 and GSK3 mutants maintained slightly higher survival rates of around 75% and 80%, respectively (Fig. 2B). In response to RA, survival rates uniformly recovered to above 90% across all genotypes, indicating a strong recovery of cold hardiness after the second exposure.

Because survival rate does not reflect plant damage or regrowth capacity, we quantified the post-freezing recovery of plant canopy. Post freezing acquired (D1and D14); top-view images were utilized to measure the absolute changes in green cover Δcover and a headroom-corrected recovery index (detailed in methodology) (Fig. 2C). During the initial recovery phase of CA, the BIL1 mutant showed the highest median of Δcover value (Fig. 2D) and maintained a stronger headroom recovery index compared with both Col-4 and GSK3 (Fig. 2E). The most significant differences occurred duirng DA. The wild-type Col-4 showed exceptionally poor canopy regrowth, as evidenced by the lowest median Δcover and a severely reduced headroom index (~ 20%) (Fig. 2D). In contrast, both mutant lines, BIL1 and GSK3, demonstrated strong post-freezing canopy recovery during DA phase. The BIL1 and GSK3 mutant achieved the highest headroom index (~ 80%) (Fig. 2E), suggests that the BR-signaling mutants confer an enhanced capacity for vegetative regrowth following the loss of cold hardiness. Furthermore, in response to RA phase, Δcover was notably lower across all genotypes (Fig. 2D), likely due to the plants already having extensive canopy cover, leaving less physical space for new expansion. However, the headroom index (Fig. 2E) revealed that all genotypes retained a strong relative capacity to recover from freezing, with the mutants exhibiting median headroom recovery rates comparable to or slightly exceeding those of Col-4.

### Multivariate analysis of chlorophyll fluorescence parameters

**Fig. 3:**
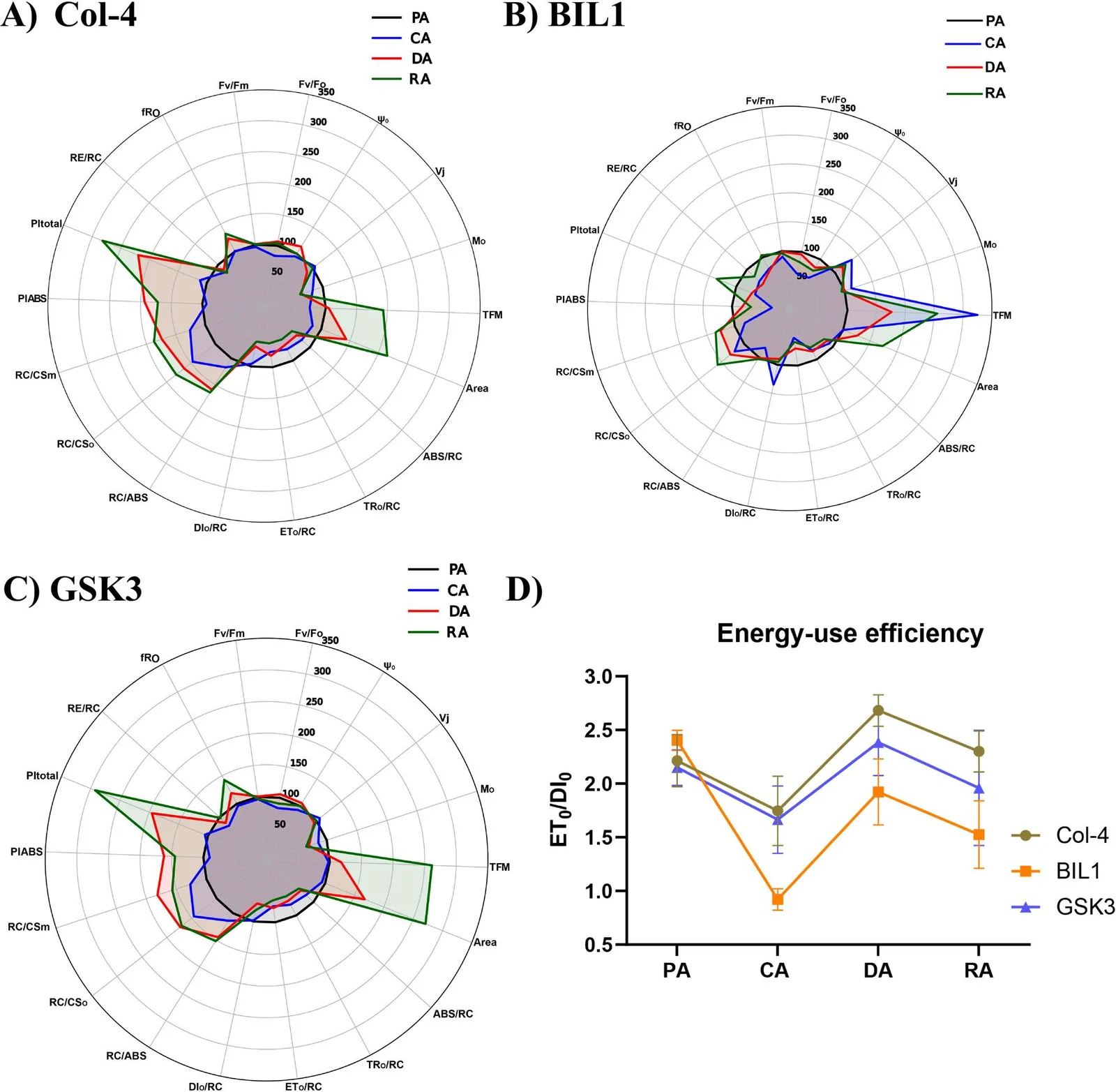
Radar plot analysis of JIP-test parameters and genotype-specific variation in energy-use efficiency under PA, CA, DA and RA phases: Radar plots showing the relative changes in key chlorophyll fluorescence and PSII performance parameters in **A** Col-4, **B** BIL1, and **C** GSK3 under PA, CA, DA and RA phases. Values are expressed relative to the PA control. **D** ET₀/DI₀ ratio across PA, CA, DA, and RA in Col-4, BIL1, and GSK3, Points represent means and error bars indicate variation among biological replicates

Multivariate analysis of chlorophyll‑fluorescence indices, energy‑flux parameters and physiological traits in response to PA, CA, DA and RA revealed distinct acclimation trajectories for Col‑4 (WT), BIL1 and GSK3 (Fig. 3A-D). The PSII and energy-flux parameters showed genotype-specific trends in response to treatments. The Col-4 during CA energy energy-dissipation indices (e.g., DI₀/RC, ) increases associated with elevated fluxes per reaction centre (ABS/RC) while reduction in several photosynthetic performance parameters (PIABS, PItotal, tF_M_, Area, φRo and ET_0_/RC); however, the maximum quantum yield of PSII (Fv/Fm) remains constant (Fig. 3A). During the DA phase, Col-4 exhibits partial recovery of photosynthetic activity, reflected by increases in Fv/Fo, ψ₀, PIABS, PItotal and several reaction-centre-related traits. The RA phase shows the strongest recovery, with marked improvements in tF_M_, Area, PI_ABS_, PI_total_, RC/CSo and RC/CSm. Although some of these parameters also increase during DA compared with CA, they do not fully return to pre-acclimation levels. BIL1 genotype showed weak photosynthesis recovery across all treatment relative to Col-4 (Fig. 3B). However, the only prominent effect was increased in tF_M_ under CA whereas all other parameters were less coordinated and although during DA and RA phase increased slightly in reaction centre related traits (PI_ABS_ and PI_total_); overall, it showed weaker recovery than Col-4 and GSK3. GSK3 shows a similar pattern to Col‑4 during CA and DA, but its recovery during RA is much stronger. In the RA phase, GSK3 displays clear increases in TFM, Area, PItotal, RC/CSo and RC/CSm, indicating a good recovery of photosynthetic performance. This strong recovery is also consistent with its higher regrowth percentage (Fig. 3C).

Furthermore, the radar-plot genotype specific differences were supported by the photosynthetic energy-use efficiency patterns observed in all three genotypes across the four treatments (Fig. 3D). The ET₀/DI₀ ratio decline in response to CA in all three genotypes increases in response to DA and RA among all genotypes. However, among the three genotypes, BIL1 shows the greatest reduction in the ET₀/DI₀ ratio, indicating a less favourable balance between electron transport and energy dissipation compared with Col-4 and especially GSK3 which showed strongest recovery. In addition, hierarchical clustering and correlation analyses were performed to identify genotype‑specific response patterns across the treatments (Figure S2A-B). The hierarchical clustering grouped Col-4 and GSK3 more closely, especially during the DA and RA phases, whereas BIL1 showed a more distinct response profile. In addition, the tissue‑level traits (RWC, OSM and LT₅₀) also separated the treatment groups, indicating that the genotype‑specific differences are not driven solely by fluorescence‑based traits but are also associated with leaf hydration status, osmotic adjustment and freezing tolerance (Figure S2A). Furthermore, the correlation matrix supported these results by showing that the fluorescence-based traits behaved in a coordinated manner, rather than independently, across all phases in all genotypes (Figure S2B). In line with these results, PIABS, RC/ABS, ψ₀ and ET_0_/RC showed strong positive correlations in GSK3, whereas these relationships were weak or inconsistent in BIL1. The tissue‑level parameters also showed positive correlations with several fluorescence‑based traits, indicating coordinated regulation and genotype‑specific response patterns.

### Transcript profiling and identification of DEGs in response to acclimation and recovery phases

Detailed transcriptomic analyses were performed for Col-4 (WT), BIL1 and GSK3 in Arabidopsis in response to CA, DA and RA treatments to investigate the acclimation mechanisms underlying the physiological changes described above. A total of 18 samples (3 genotypes × 3 treatments × 2 replicates) were obtained, and each sample produced clean data with a minimum yield of 3.06 GB and more than 92% of bases achieving a Q30 score or higher. The clean reads were mapped to the reference genome, and a total of 32,833 genes were annotated and analysed. Principal component analysis (PCA) and hierarchical clustering of the top 500 most variable VST-transformed genes were performed (Figure S3). PCA showed 72.9% of the total variance through the first two components (PC1 = 43.6% and PC2 = 29.3%) and clearly separated the genotypes and treatments (Figure S3A). Moreover, Col-4 and GSK3 showed more similar distributions along PC1 across treatments, whereas BIL1 diverged more strongly along PC2, indicating that its transcriptomic responses to CA, DA and RA were more distinct. Furthermore, hierarchical clustering of the top 500 genes as the global expression supported these results, showing that Col‑4 and GSK3 exhibited more similar transcriptional trends across treatments than BIL1 (Figure S3B).

**Fig. 4:**
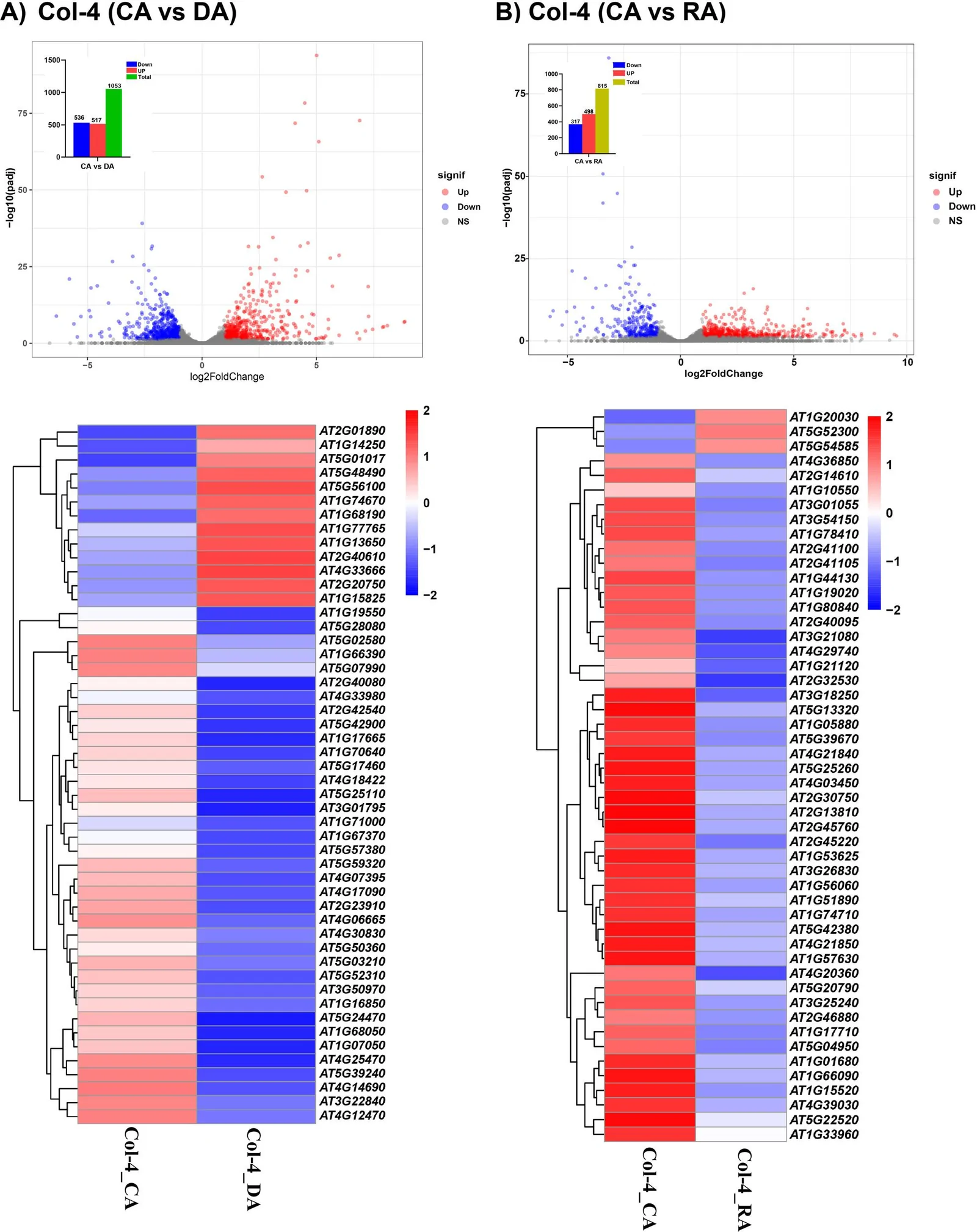
Differential gene expression patterns in Col-4 **A**) CA vs DA and **B**) CA vs RA: In each volcano plot, red and blue points represent significantly upregulated and downregulated genes, respectively. The inset bar charts summarize the numbers of upregulated, downregulated, and total differentially expressed genes for each comparison. Heatmaps display the expression patterns of selected top 50 DEGs across the treatment pairs. Expression values are shown as row-scaled Z-scores, where red indicates relatively higher expression and blue indicates relatively lower expression

**Fig. 5:**
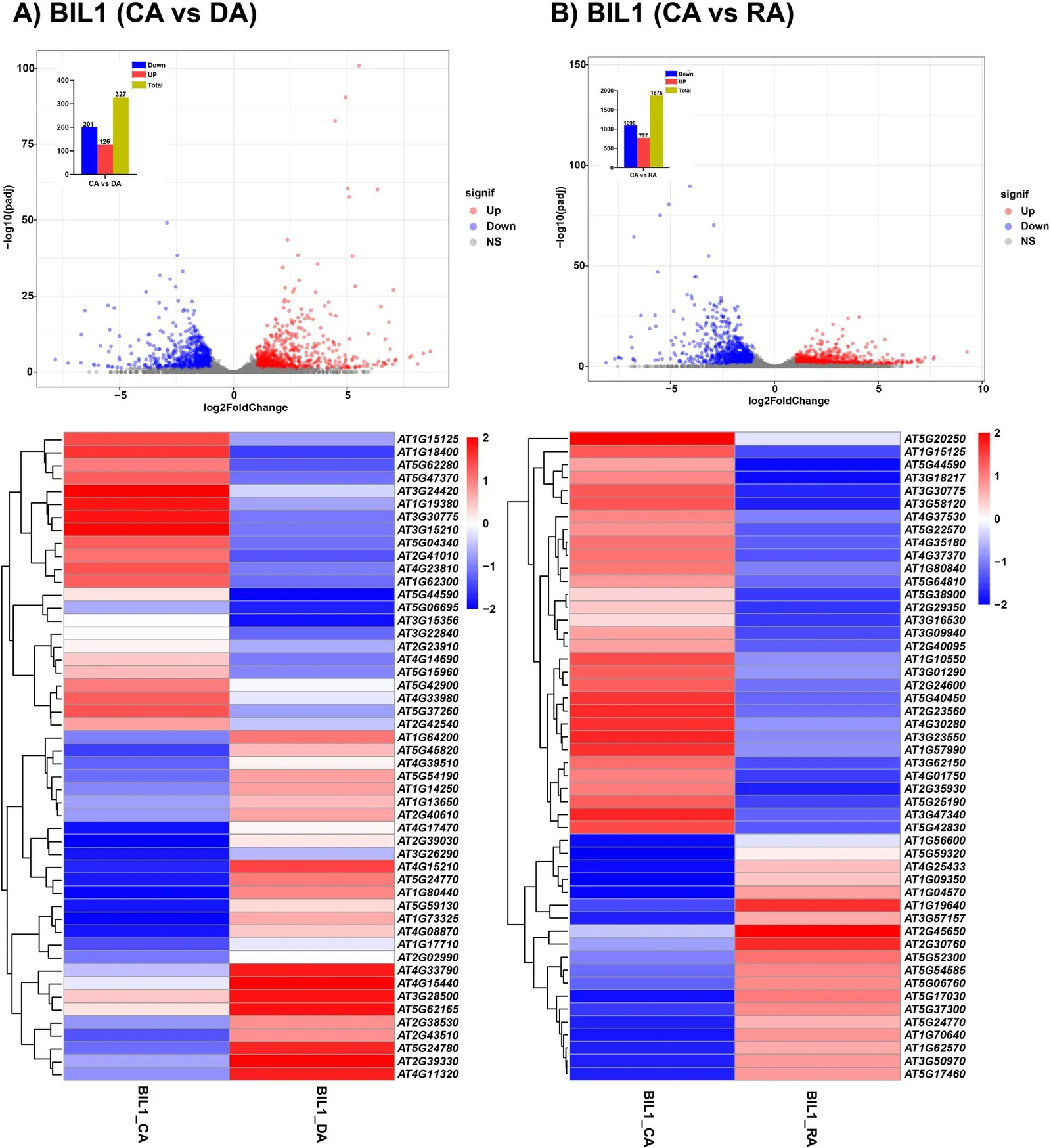
Differential gene expression patterns in BIL1 **A**) CA vs DA and **B**) CA vs RA: In each volcano plot, red and blue points represent significantly upregulated and downregulated genes, respectively. The inset bar charts summarize the numbers of upregulated, downregulated, and total differentially expressed genes for each comparison. Heatmaps display the expression patterns of selected top 50 DEGs across the treatment pairs. Expression values are shown as row-scaled Z-scores, where red indicates relatively higher expression and blue indicates relatively lower expression

**Fig. 6:**
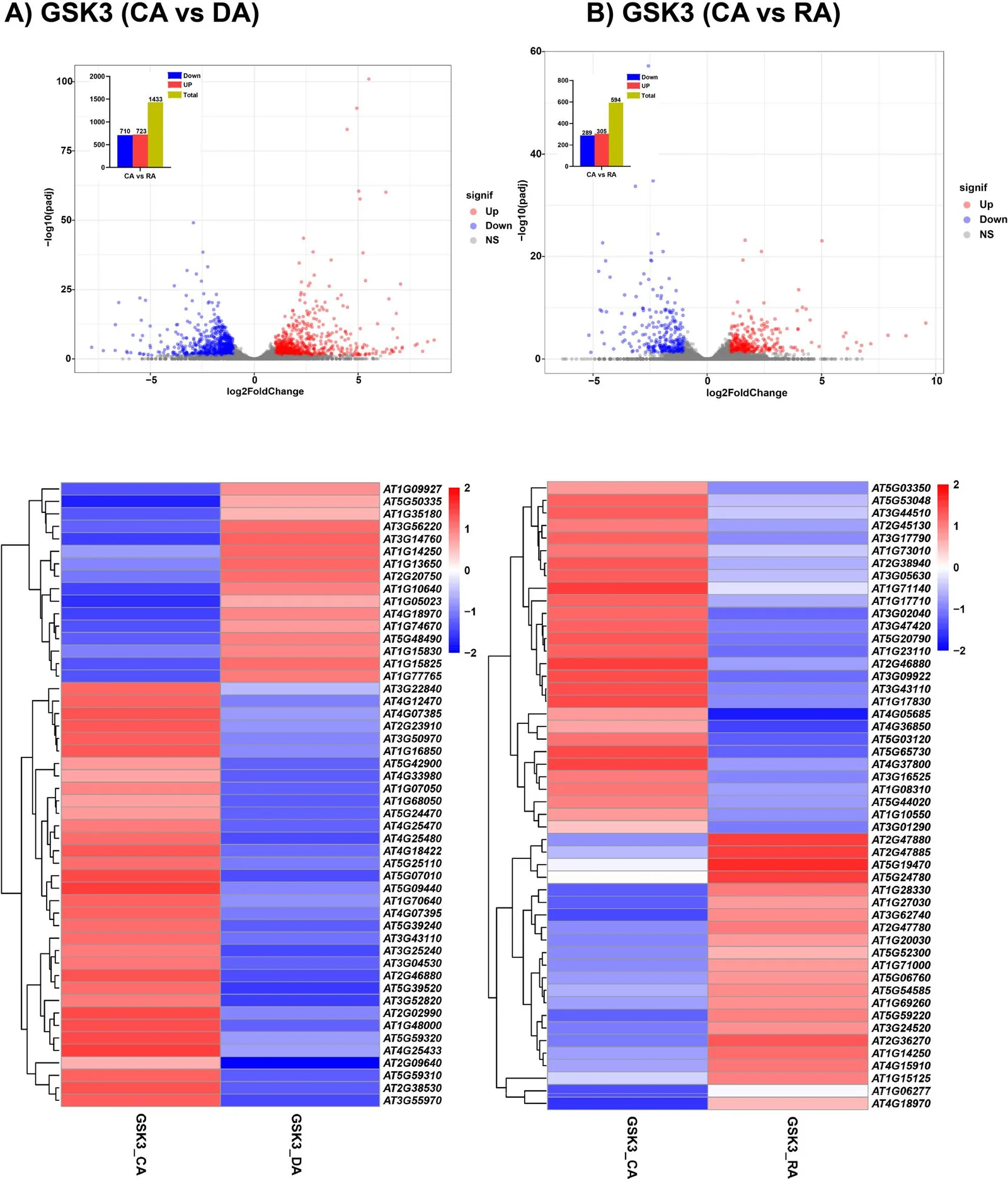
Differential gene expression patterns in GSK3 **A**) CA vs DA and **B**) CA vs RA: In each volcano plot, red and blue points represent significantly upregulated and downregulated genes, respectively. The inset bar charts summarize the numbers of upregulated, downregulated, and total differentially expressed genes for each comparison. Heatmaps display the expression patterns of selected top 50 DEGs across the treatment pairs. Expression values are shown as row-scaled Z-scores, where red indicates relatively higher expression and blue indicates relatively lower expression

Furthermore, differentially expressed genes (DEGs) were identified in response to CA, DA and RA in Col-4, BIL1 and GSK3. Based on the physiological responses and initial mapping results, the following comparisons were selected to identify DEGs: Col-4 (CA vs. DA, CA vs. RA), BIL1 (CA vs. DA, CA vs. RA) and GSK3 (CA vs. DA, CA vs. RA). In the CA vs. DA comparison, GSK3 exhibited the largest transcriptional response, with 1,433 DEGs (723 up‑regulated and 710 down‑regulated), followed by Col‑4 with 1,053 DEGs (517 up‑regulated and 536 down‑regulated). In contrast, BIL1 showed a much smaller response, with only 327 DEGs (126 up‑regulated and 201 down‑regulated) (Figs. 4 and 5, 6 A). However, in the CA vs. RA comparison, BIL1 showed the strongest divergence, with 1,876 DEGs (777 up-regulated and 1,099 down-regulated), whereas Col-4 and GSK3 exhibited much smaller responses, with 815 DEGs (498 up-regulated and 317 down-regulated) and 594 DEGs (305 up-regulated and 289 down-regulated), respectively (Figs. 4B, 5 and 6B). These results indicated that de‑acclimation causes a weaker disruption in BIL1 compared with Col‑4 and GSK3, whereas re‑acclimation restores a stronger CA‑like transcriptional state in Col‑4 and GSK3 than in BIL1. The heatmap of top DEGs supported this pattern and showed clear mutual expression shifts between the genotypes and treatments (Figs. 4 and 5, 6). Likewise, the broader heatmap of up‑regulated and down‑regulated genes across all genotypes further confirmed that Col‑4 and GSK3 shared more similar expression patterns than BIL1, particularly during the re‑acclimation phase (Figure S4-S9).

There were also subsets of DEGs that showed overlapping and unique responses among the genotypes and treatments. A total of 329 up-regulated and 425 down-regulated genes were common to all three genotypes (Figure S10A-B). Moreover, 244, 161 and 328 up-regulated genes and 107, 311 and 222 down-regulated genes were unique to Col-4, BIL1 and GSK3, respectively. Moreover, pairwise overlap was larger between Col-4 and GSK3, which shared 402 up-regulated and 251 down-regulated DEGs with each other, than BIL1. This indicated that Col-4 and GSK3 responded more similarly to treatment then BIL1. At specific comparison level (CA vs. DA and CA vs. RA), the DEG overlap showed the same pattern, with Col-4 and GSK3 sharing more similar DEGs in both up and down regulation than BIL1, which showed more divergent responses (Figure S11A-C). This pattern suggests that transcriptomic reprogramming during de-acclimation and re-acclimation is strongly genotype-dependent. Moreover, these results align with the physiological responses of all three genotypes to CA, DA and RA. To further understand the biological relevance of these expression patterns, Gene Ontology (GO) and KEGG pathway analyses were performed on the identified DEGs.

### Functional enrichment and KEGG pathway analyses of differentially expressed genes

GO enrichment analysis classified the identified DEGs into three major categories: biological process (BP), cellular component (CC), and molecular function (MF) (Figure S12-14). In Col-4, the up-regulated DEGs were most enriched in stress- and acclimation-related biological processes, including response to water, water deprivation, cold, oxygen limitation, defense response, and photosynthesis-associated functions, including light reaction and tetrapyrrole-related metabolism in both comparison CA vs. DA and CA vs. RA. In contrast, the down-regulated DEGs were enriched in chlorophyll biosynthesis, tetrapyrrole and porphyrin-containing compound metabolism, photosynthesis, and light-harvesting functions; with stronger enrichment in CA vs. DA than in CA vs. RA (Figure S12). In the cellular component category, both up‑ and down‑regulated DEGs were predominantly enriched in chloroplast, thylakoid, plastid thylakoid membrane, and photosystem‑related structures. In the molecular function category, enriched terms included chlorophyll binding, water channel activity, UDP‑glycosyltransferase activity, and various oxidoreductase‑related functions.

In BIL1, up-regulated genes in both comparisons were enriched in biological processes related to defense- and stress-related biological processes. In contrast, down-regulated genes were enriched in rRNA metabolic process, sulfur compound metabolism, pigment metabolism, circadian and rhythmic processes, and multiple stress-related categories, including cold acclimation, response to cold, response to water, and response to water deprivation. However, in the CA vs. DA comparison, both the up- and down-regulated DEGs displayed distinct enrichment patterns than CA vs. RA (Figure S13). In the cellular component and molecular function categories, both comparisons showed similar enrichment patterns. The up‑regulated DEGs were enriched in chloroplast‑ and plastid‑associated structures (CC) and membrane‑associated signaling functions (MF). In contrast, the down‑regulated DEGs were enriched in terms related to the repression of ribosome‑ and plastid‑associated cellular structures (CC), as well as reduced core plastid‑ and ribosome‑related (MF) (Figure S13). In GSK3, both the CA vs. DA and CA vs. RA comparisons were strongly enriched in photosynthesis- and chloroplast-related functions, although the enrichment was markedly stronger in CA vs. DA. This indicates that de-acclimation caused a greater divergence from the cold-acclimated state than re-acclimation (Figure S14). Consistently, the up and down-regulated DEGs were enriched in biological processes associated with photosynthetic reorganization, in chloroplast-related cellular components, and in molecular functions linked to enzymatic activity and transport. Overall, these results indicate that Col-4 exhibits a mixed and moderately coordinated response, BIL1 shows a weak recovery pattern, and GSK3 displays a strong photosynthetic and chloroplast-associated response. However, the signal was substantially stronger in the CA vs. DA comparison than in CA vs. RA, indicating that re-acclimation only partially restored the cold-acclimated state.

**Fig. 7:**
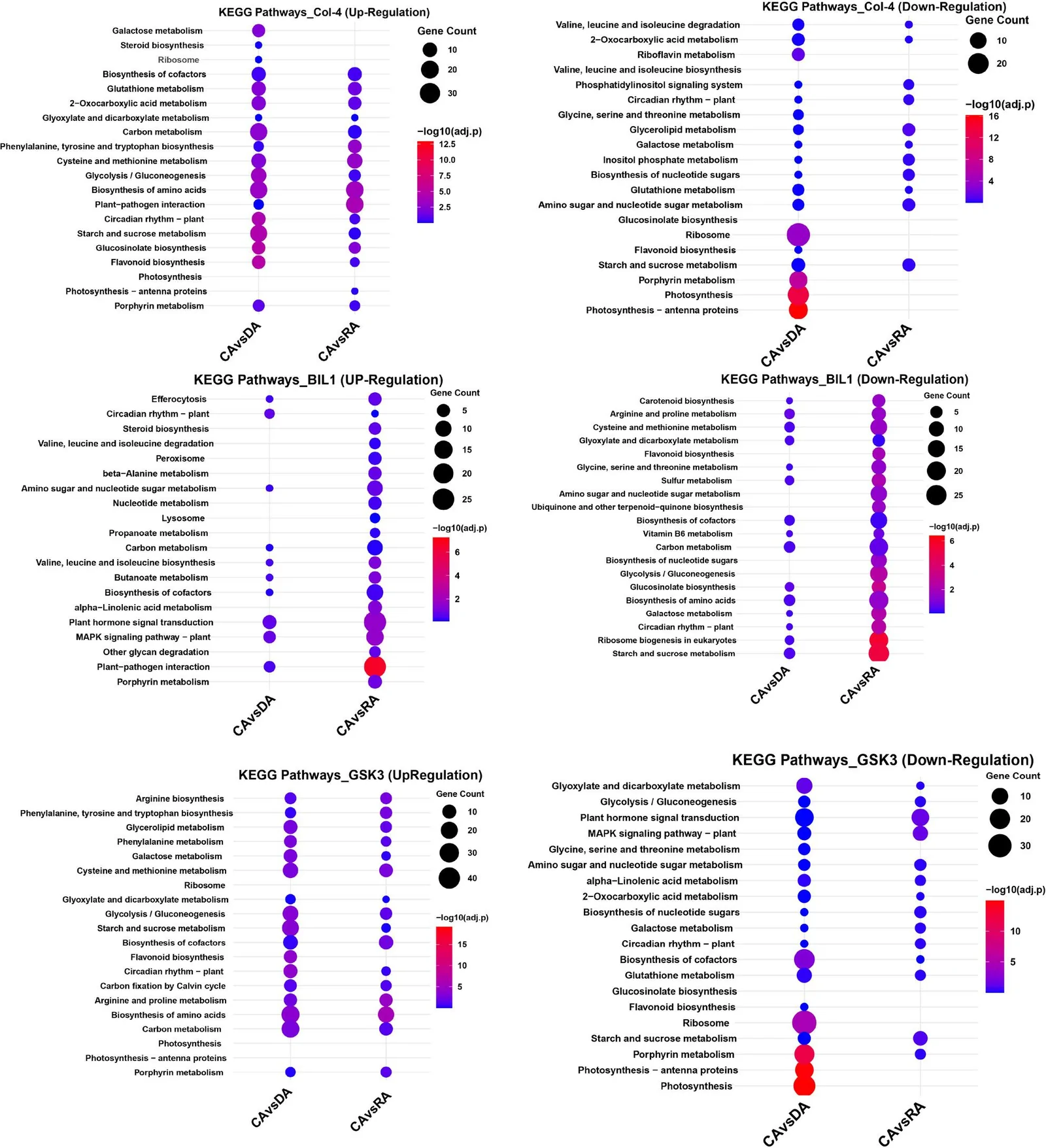
KEGG pathway enrichment of upregulated and downregulated genes in Col-4, BIL1, and GSK3 during de-acclimation and re-acclimation processes. Bubble plots showing significantly enriched KEGG pathways for upregulated and downregulated differentially expressed genes in Col-4, BIL1, and GSK3 for the comparisons CA vs DA and CA vs RA. Each bubble represents one enriched pathway; bubble size indicates the number of genes assigned to that pathway, and bubble colour represents enrichment significance as −log10(adjusted *p*-value)

Additionally, KEGG pathway analysis confirmed the above findings for the de-acclimation and re-acclimation processes. Col-4 had an intermediate profile, showing a high level of carbon metabolism, amino acid biosynthesis, glycolysis/gluconeogenesis, and other stress-related metabolic pathways, as well as a down-regulation of photosynthesis, antenna proteins, and porphyrins, especially for CA vs. DA (Fig. 7). BIL1 showed more significant pathways of stress and signaling-related, including plant-pathogen interaction, MAPK signaling, and plant hormone signal transduction, as well as down-regulation of biosynthetic and metabolic processes. GSK3 had the highest level of coordination for photosynthesis-related processes, including photosynthesis, photosynthesis-antenna proteins, porphyrins, carbon metabolism, amino acid biosynthesis, especially for CA vs. DA, indicating a more significant response during de-acclimation than during re-acclimation. Overall, a distinct hierarchy of genotypes is evident, where GSK3 had the highest level of reorganization and recovery of photosynthesis (Fig. 7).

Moreover, we extracted the expression profiles of canonical cold-response regulators (*ICE1/SCRM*, *CBF1*–*CBF3/DREB1B*-*A*, *COR15A*) and core BR-signalling components (*BZR1*, *BES1/BZR2*, *BIN2*) from the FPKM data matrix (Figure S15, Table S1). As expected, The *CBF*-*COR* module showed the expected dynamics coordination during CA, establishing the transcriptional baseline for subsequent phases. In Col-4 and GSK3, *CBF1*-*3* and *COR15A* were down-regulated during DA and up-regulated during RA. Notably, *COR15A* accumulated to ~3-fold above its CA level, and *CBF3* exceeded its CA expression in both genotypes, indicating a robust re-activation of the *ICE1-CBF-COR* cascade in response to RA. In contrast, the regulatory dynamics of the CBF-module were highly disrupted in BIL1, as it failed to down-regulate during DA and, not recovered *CBF1* expression during RA, although *COR15A* up-regulated was retained. This pattern indicates a partial decoupling of the *ICE1-CBF-COR* cascade in the absence of functional BIL1 (Figure S15). *ICE1*, *BZR1*, *BES1* and *BIN2* showed minimal transcriptional change across all genotypes in response to CA, DA and RA treatments, indicating their post-translational regulation. The targeted kinase loci confirmed disruption of the insertion: *At2g30980* (*BIL1*) transcript was reduced approximately ~3-fold in the BIL1 mutant relative to Col-4 in response to RA, and *At1g06390* (*GSK3*) was reduced by approximately 40–60% in the GSK3 mutant across treatments (Table S1). 

### Co-expression network analysis in BR‑signalling mutants of *Arabidopsis*

Co-expression network analysis (WGCNA) was performed to visualize genotype-associated co-expression patterns during de-acclimation and re-acclimation, which provides additional evidence that coordination to the physiological responses, DEG profiles, and functional enrichment findings. The gene dendrogram and module color relationship showed clear partitioning of transcripts into distinct co‑expression modules (Figure S16A), while the soft‑thresholding analysis supported the selected network power required to approximate scale‑free topology (Figure S16B). Furthermore, the network heatmap confirmed strong intramodular connectivity, indicating biologically coherent co‑expression structures (Figure S16C). This structure was further supported by the module–traits relationship analysis, which incorporated genotype and its interaction with treatment. The analysis revealed clear and distinct associations for Col‑4, BIL1, and GSK3 across the CA, DA, and RA conditions (Figure S16D).

Among the module–trait relationships, the purple module in BIL1 (CA vs. RA) and the light‑yellow module in GSK3 (CA vs. RA) showed the strongest associations. These two modules were therefore selected for downstream hub‑gene network construction and functional enrichment analysis. The hub‑gene network for purple module of BIL1 (CA vs. RA) formed a smaller and less dense connected network with very few central hub genes (*AT1G04160*,* AT1G20090*,* AT1G12430*,* AT1G08660*,* AT1G01960*,* and AT1G07950*) (Figure S17A). In contrast, the light-yellow module in GSK3 (CA vs. RA) formed a very strong and highly interconnected co-expression network, indicating extensive transcriptional reprogramming during the re-acclimation phase in this genotype (Figure S17B). Functional enrichment analysis of these modules further distinguished their biological roles. The purple module in BIL1 exhibited a divergent functional profile compared with the light-yellow module in GSK3, highlighting clear differences in the biological processes underlying their respective co-expression patterns (Figure S17C-D). GSK3 showed a more coordinated metabolic and signalling-related recovery network, reflecting its greater capacity to response during RA process.

## Discussion

The present study investigated the physiological and transcriptomic responses of *Arabidopsis thaliana* wild‑type Col‑4 together with two brassinosteroid‑signaling mutants carrying T‑DNA insertions, *AtBIL1* (*At2g30980*) and *AtGSK3* (*At1g06390*), a member of the GSK‑like kinase family, across the CA, DA, and RA phases (Figure S1). Overall, results show how disturbances in the brassinosteroid (BR) signalling pathway influence both plant architecture and gene expression during cold acclimation and re‑acclimation processes. The combined physiological and transcriptomic evidence revealed strong genotype-specific responses, highlights that GSK-like kinase activity may act as primary; yet functionally diversified regulator of cold resilience, suggesting that *Arabidopsis* can serve as a useful model for improving cold tolerance in crops.

### Physiological insight in response to cold treatment and post-freeze recovery of BR signalling mutant

Low RWC reaching approximately ~ 55% across all genotypes (wild type and two BR-signalling mutants) in response to CA is consistent with cold-induced cellular dehydration. Physiological adjustments involving controlled dehydration prepare cells for subsequent freezing. BRs are well established as drivers of cell elongation, cell division, and meristematic activity: BIN2 phosphorylates and negatively regulates BZR1 and BES1, whereas their nuclear accumulation promotes the transcription of genes required for cell expansion and growth [33–37], and is further supported by cold-induced reductions in membrane permeability and water channel activity slowing water distribution across cellular compartments [1, 38]. During the DA and RA, all three genotypes recovered their water content, showing clear increase compared with the CA phase (Fig. 1A). The *AtGSK3* mutant showed the highest level of hydration compared to Col-4 and BIL1 in DA phase suggesting that mutation in *At1g06390* (GSK-kinase activity) alters water status adjustment kinetics without permanently affecting water retention capacity, consistent with the known roles of group III GSK-like kinases in ABA dependent stomatal and hydraulic signalling [39, 40]. During the RA phase, all genotypes showed similar RWC values (~ 70%) (Fig. 1A), which may be consistent with a form of physiological priming established during the first cold exposure that could facilitate faster water balance adjustment during RA. It is usually observed that plants often respond more effectively to repeated stress than to the initial exposure and showed improved adjustment and recovery [9, 41].

The osmotic responses showed variation (genotype specific response) during CA. The BIL1 mutant showed a stronger osmotic adjustment (− 2.6 MPa) compared with Col‑4 and GSK3 (Fig. 1B). Osmotic adjustment refers to the active accumulation of compatible solutes as well as soluble sugars (sucrose, glucose, fructose) during water deficit, which lowers cellular water potential, helps to maintain turgor pressure, and protects macromolecules from dehydration damage [42]. In many plants, including *Arabidopsis* and rapeseed, both cold and light are required to induce the accumulation of sugars and proline needed for freezing resistance, linking photosynthetic carbon supply directly to osmotic defence. The strong osmotic adjustment observed in the BIL1 mutant is therefore likely driven by elevated compatible‑solute biosynthesis resulting from altered BR signalling pathway [43, 44]. During the DA and RA phases, OSM values returned to wild-type levels across all genotypes (Fig. 1B), indicating that the enhanced osmotic adjustment is a transient, phase-specific response rather than a constitutive trait [33, 35, 45]. Moreover, osmotic adjustment lowers cellular osmotic potential and thereby limits freeze‑induced dehydration, while accumulated compatible solutes stabilise membranes and proteins and buffer ROS. These combined effects provide a mechanistic link between osmotic adjustment and freezing tolerance. However, osmotic adjustment is only one facet of freezing tolerance: despite showing the strongest osmotic adjustment during CA, BIL1 did not exhibit the greatest re‑hardening, indicating that solute accumulation alone cannot maximise freezing tolerance and aligns with the growth-defence trade‑off.

Furthermore, the freezing tests and post‑freezing recovery also revealed genotype‑specific responses, likely reflecting the effects of their insertion mutations, which alter the BR‑signalling pathway. Lethal temperature (LT₅₀) showed more significant genotype dependent response of re-acclimated plants (Fig. 2A). All genotypes achieved comparable freezing tolerance in response to CA and similar response to hardiness during DA. However, their RA responses diverged distinctly: Col-4 reached LT₅₀ ≈ −20 °C, whereas the BIL1 and GSK3 mutants exhibited more moderate recovery (− 15 °C and − 13 °C, respectively) (Fig. 2A). This divergence is mechanistically coherent given the established role of *BIN2/BIL1* in regulating the stability of *ICE1*, the master transcription factor driving *CBF-*dependent cold-responsive gene expression. The *BIN2* phosphorylates *ICE1* under prolonged cold, promoting HOS1-mediated proteasomal degradation and attenuating CBF expression during sustained stress [18, 46]. As *BIL1* is a paralogue rather than BIN2 itself, the mechanistic links drawn from *BIN2* to the *BIL1* mutant reflect assumptions of functional similarity within the GSK3/shaggy‑like kinase clade and should be investigated for *BIL1*.The transient suppression of *BIN2* activity during early cold exposure creates a temporal window of maximal *ICE1–CBF* activity that is essential for establishing freezing tolerance. Loss of *BIL1* may compresses this regulatory window, reducing the amplitude of CBF‑driven re‑hardening during RA. This response aligns with BR‑signalling‑defective mutants are hypersensitive to freezing, whereas constitutive BR activation enhances cold resistance through both CESTA‑mediated CBF induction and CBF‑independent COR gene regulation [20]. Similarly, transcriptomic data show that the *CBF*-*COR* module is dismantled during DA and recovered during RA in Col-4 and GSK3, whereas BIL1 fails to restore *CBF1* during RA (Figure S15). The transcriptional stability of *ICE1*, *BZR1* and *BES1* indicates that the BR–cold interactions may exert post-translationally; for example, via *BIN2*-dependent *ICE1* phosphorylation and stability, rather than through changes in marker-gene transcript abundance.

The post-freeze canopy recovery data revealed a significant variation: despite exhibiting lower freezing tolerance (higher LT₅₀ values) during DA, the mutants showed dramatically superior vegetative regrowth following freezing at this stage. Col-4 displayed the poorest canopy recovery (headroom index ~ 20%), whereas both BIL1 and GSK3 achieved recovery indices of approximately 80% (Fig. 2D-E). This dissociation between intrinsic hardiness and post‑freeze recovery capacity has important ecological implications and points to a mechanistically distinct role for GSK‑like kinases in vegetative regrowth. BRs are well established as drivers of cell elongation, cell division, and meristematic activity: BIN2 phosphorylates and negatively regulates BZR1 and BES1, whereas their nuclear accumulation promotes the transcription of genes required for cell expansion and growth [33, 34]. During the post-freeze recovery period, when cells damaged by ice formation must be replaced through new growth from surviving meristematic zones, this constitutively elevated growth signalling may provide a substantial advantage by accelerating cell elongation and division in the expanding canopy. Moreover, BIN2 has been identified as a critical decision-making regulator in seedlings exposed to combined abiotic stresses, with the BIN2 clade of shaggy-like kinases required for appropriate differential allocation of growth resources between organs under adverse conditions [47]. Loss of this regulatory capacity in the mutants may paradoxically liberate growth potential under post-freeze conditions, where the primary constraint shifts from stress protection to rapid tissue reconstruction. Overall survival remained high across all genotypes (> 80%), with Col‑4 declining to ~ 65% during DA while the mutants maintained ~ 75–80% (Fig. 2B). This pattern further supports a growth–defence trade‑off, whereby constitutive BR‑pathway activation enhances post‑freeze resilience at the expense of maximal re‑hardening capacity [20].

Multivariate JIP-test analysis revealed genotype-specific photosynthetic reorganisation across treatment phases. During CA, all genotypes exhibited suppressed PSII efficiency, characterised by increased energy dissipation (DI₀/RC), reduced electron transport (ET₀/RC), and elevated absorption per reaction centre (ABS/RC) (Fig. 3A-C) which is a pattern consistent with cold-induced photoinhibition, where chilling disrupts the D1-protein repair cycle and creates an imbalance between PSII damage and recovery [48–51]. GSK3 exhibited the strongest photosynthetic efficiency, showing the largest values in TFM, Area, Ptotal, RC/CSo, and RC/CSm, indicative of restored PSII connectivity and enhanced electron‑transport capacity (Fig. 3C). In contrast, BIL1 displayed the weakest efficiency, with a persistently low ET₀/DI₀ ratio (Fig. 3B, D), reflecting an unfavourable balance between productive electron transport and thermal dissipation arising from insufficient transcriptional reprogramming of photosynthetic components [50]. The consistent clustering of Col-4 and GSK3 separately from BIL1 suggests that impaired photosynthetic efficiency is specifically associated with BIL1 kinase function, possibly through its regulation of chloroplast signalling components [52, 53]. The positive correlation between photosynthetic efficiency (GSK3 > Col-4 > BIL1) and vegetative regrowth showed strong association between PSII restoration and whole-plant resilience, consistent with the role of photosynthetic assimilates as osmolytes and cryoprotectants during cold acclimation [35, 54]. Together, the coordinated behaviour of fluorescence traits and tissue-level parameters (RWC, OSM, LT₅₀) demonstrates that the genotype-specific stress responses observed in the present study reflected strong multi-trait phenotypic differences rather than isolated photosynthetic changes.

### Transcriptomic reprogramming of DEGs and their functional annotations

The differentially expressions gene (DEG) count data exhibited a clear reciprocal pattern of transcriptional regulation between the CA vs. DA and CA vs. RA comparisons. During CA vs. DA, GSK3 showed the strongest transcriptional response (1,433 DEGs) (Fig. 6A), followed by Col‑4 (1,053) (Fig. 4A) and BIL1 (327) (Fig. 5A). In contrast, during CA vs. RA this hierarchy reversed: BIL1 exhibited the largest reprogramming (1,876 DEGs) (Fig. 5B), whereas Col‑4 and GSK3 showed far fewer changes (815 and 594 DEGs) (Figs. 4B and 6B). This trend is biologically significant, as the small DA response in the BIL1 mutant suggests a delayed or weakened ability to downregulate cold‑acclimation programmes and activate growth‑associated transcription when temperatures return to warmer conditions. De‑acclimation is a tightly regulated transition requiring rapid hormonal adjustments particularly in auxin, gibberellin, and ABA signalling, and an early surge in translational activity driven by rapidly accumulating ribosomal proteins [55, 56]. The reduced responsiveness of BIL1 to these hormonal and translational cues likely reflects a restricted dynamic range in BR-ABA crosstalk, as *BIN2*, and by interference BIL1, kinase activity integrates these pathways by phosphorylating both BZR1/BES1 and ABA‑signalling components [33]. In contrast, the larger DEG count in BIL1 during the CA vs. RA comparison indicates that, despite its blunted DA response, BIL1 undergoes extensive transcriptional reconfiguration during re‑acclimation (Fig. 5B); however, this reprogramming does not effectively restore a cold‑acclimated transcriptional state. This pattern is supported by the physiological data: BIL1 showed the weakest LT₅₀ recovery during RA and the lowest photosynthetic performance indices, indicating that its extensive transcriptional activity during RA is largely functionally ineffective from re-hardening. Similar patterns have been reported in other stress contexts, where mutations in key regulatory genes trigger broad transcriptional activation without functional integration; in the present data, the genes reprogrammed in BIL1 during RA were enriched for defence- and pathogen-related pathways (e.g., plant–pathogen interaction and MAPK signalling) rather than the canonical cold-acclimation and *CBF*–*COR*-associated categories that characterised Col-4 and GSK3 (Figs. 7 and S13), indicating that this extensive reprogramming was not preferentially directed toward restore the cold-acclimated transcriptional state [9].The convergence of Col-4 and GSK3 transcriptomes similar in their larger shared DEG pools (402 up-regulated and 251 down-regulated) and their more similar PCA distributions suggests that the kinase encoded by *At1g06390* (GSK3) occupies a qualitatively different regulatory position from BIL1 within the cold-response network (Fig. 6A & B). Group‑III *GSK‑like kinases* (including GSK3) differ from the group‑II *BIN2/BIL1* clade in both substrate specificity and regulatory context. This clade includes *ASKθ/GSK3*, which participates in BR signalling and stress responses in ways that partially overlap with but are different from *BIN2* function [52, 57]. Overlap analysis confirmed that de-acclimation and re-acclimation transcriptional reprogramming are strongly genotype-dependent: the 329 up- and 425 down-regulated DEGs shared across all genotypes (Figure S10A-B); likely represent a conserved cold-responsive core, potentially including canonical COR genes under CBF/DREB1 control. In contrast, the genotype-unique DEG sets (244 in Col-4, 161 in BIL1, and 328 in GSK3) (Figure S11A-C); reflect genotype-specific transcriptional diversification driven by differential activation of BR-responsive, ABA-responsive, or hormone-independent stress-signalling pathways.

GO and KEGG enrichment analyses revealed functionally distinct signatures among the three genotypes andprovided biological context that support the numerical DEG patterns. In Col-4, up-regulated DEGs were enriched for stress- and acclimation-related processes (responses to cold, water, and defence), whereas down-regulated DEGs reflected reduced chloroplast anabolism (Figure S12) as with a coordinated shift from growth to protection during de-acclimation, as described by [55]. BIL1 showed enrichment for defence- and pathogen-related pathways (plant-pathogen interaction, MAPK signalling) in both comparisons, likely reflecting cross-activation through shared biotic and abiotic signalling components, including the ICE1-associated MAPK cascade [58, 59] (Figure S13). Down-regulated genes in BIL1 were enriched for rRNA metabolism, ribosome biogenesis, and circadian processes, suggesting impaired translational buffering, as ribosomal protein mRNAs are stored during cold and rapidly mobilised upon de-acclimation to resume growth [55], failure to maintain this reserve may underlie both the reduced DA response of BIL1 and its poor photosynthetic recovery during RA.

GSK3 displayed the most coherent and coordinated functional enrichment signature, dominated by photosynthesis‑related KEGG pathways: photosynthesis, antenna‑protein complexes, porphyrin metabolism, and carbon metabolism were strongly enriched in both up‑ and down‑regulated DEGs, with markedly stronger enrichment in the CA vs. DA comparison than in CA vs. RA (Fig. 7 & S14). This pattern suggests that GSK3 undergoes a more substantial transcriptional reorganisation of its photosynthetic activity during DA than RA, consistent with more efficient re‑establishment of the cold‑acclimated chloroplast state during RA. Because the photosynthetic machinery retains a memory of its cold‑adapted configuration, fewer transcriptional adjustments are required during RA. These results suggest that plant exhibit a form of transcriptional events, in which prior cold exposure reinforces the responsiveness of stress‑activated genes during subsequent recovery phases [60]. The additional enrichment of carbon metabolism, amino acid biosynthesis, and glycolysis/gluconeogenesis in GSK3 further indicates that this genotype achieves a more globally coordinated metabolic reorientation during de-acclimation, adjusting photosynthetic output, carbon assimilation, and biosynthetic flux more effectively than the fragmented metabolic response observed in BIL1.

WGCNA co‑expression analysis further supported the genotype‑specific hierarchy underlying the physiological and transcriptomic responses (Figure S15). Module-trait association identified the purple module in BIL1 (CA vs. RA) (Figure S16A) and the light-yellow module in GSK3 (CA vs. RA) (Figure S16B), as the strongest genotype treatment associations. Their contrasting network architectures are biologically informative, as GSK3 dense, highly connected light-yellow module indicates well-orchestrated transcriptional reprogramming during RA, whereas the BIL1 purple module showed regulatory fragmentation consistent with its poor physiological re-hardening. In network biology, module connectivity serves as a direct proxy for transcriptional coordination, with hub-driven dense modules typically reflecting coherent regulation by master transcription factors or signalling nodes [32, 61]. Similar hub-centric logic has been documented in cold-stress WGCNA studies in maize and cotton [62]. The hubs identified in both modules represent strong candidates for functional validation. CRISPR-based interrogation of these genes would substantially advance mechanistic understanding of how each mutant shapes the transcriptional architecture of re-acclimation.

Overall, our results reveal a broader picture of BR‑pathway function during dynamic temperature shifts associated with cold acclimation, de-acclimation and re-acclimation. The BIL1 mutant enhances osmotic adjustment during CA and supports post‑freeze regrowth during DA, yet compromises maximal freezing tolerance and photosynthetic re‑acclimation during RA. In contrast, the GSK3 mutant exhibits strong photosynthetic recovery and highly coordinated transcriptional reprogramming during RA, while also conferring enhanced post‑freeze regrowth which is a physiological advantage partially shared with BIL1. All three genotypes returned to similar RWC levels during RA, even though their freezing tolerance and gene-expression patterns behave differently. This indicates that hydraulic memory is broadly conserved, while GSK-like kinases play a more specific role in how plants re-acclimate and rebuild freezing tolerance. In natural temperate environments, cycles of cold, thawing, and re-hardening are becoming more common with climate change, and wild-type GSK-like kinase activity appears to provide the best balance between rescue freezing tolerance and resuming growth. Mutations that shift this balance create genotype-specific trade-offs, which helps to reveal how plants regulate cold resilience.

## Conclusion

The present study demonstrates that cold acclimation and re‑acclimation trigger strong but genotype‑specific physiological and transcriptional changes in Arabidopsis thaliana. Marked differences were observed between the wild type (Col‑4) and the two BR‑signalling mutants (BIL1 and GSK3). Although all genotypes experienced reduced hydration and declines in photosynthetic performance during CA, followed by partial recovery, their survival strategies diverged substantially. Col-4 exhibited the strongest recovery of freezing tolerance during RA; however, the mutant genotypes, particularly BIL1 and GSK3 showed superior regrowth following freezing injury, indicating a trade-off between maximal cold hardiness and recovery capacity. BIL1 demonstrated strong osmotic adjustment but weak and poorly coordinated recovery of photosynthesis and transcriptomic regulation, whereas GSK3 showed more coherent photosynthetic restoration and tighter regulation of gene expression. Moreover, transcriptomic and functional enrichment analyses revealed that Col-4 and GSK3 share more integrated and functionally coordinated transcriptional profiles, while BIL1 displays a more fragmented regulatory network. Together, these findings suggest that GSK-like kinases help regulate the balance between growth, stress tolerance, and recovery from freezing injury, shifting plants between these states in response to environmental conditions.

## Supplementary Information

Supplementary Material 1: Table S1. Mean FPKM values and log₂ fold-changes of canonical cold- and BR-signalling genes.

Supplementary Material 2: Figure S1. Representative visual phenotypes of Arabidopsis thaliana genotypes Col‑4 (WT), BIL1, and GSK3 across acclimation treatments: PA (21 days after sowing), CA (31 days after sowing), DA (35 days after sowing), and RA (38 days after sowing).

Supplementary Material 3: Figure S2. Multivariate analysis of physiological traits across genotypes in response to acclimation treatments. A) Heatmap showing the clustering of Col‑4, BIL1, and GSK3 plants under CA, DA, and RA treatments based on chlorophyll fluorescence, water status, and stress‑related parameters. B) Correlation analysis illustrating pairwise relationships among all measured physiological traits, with positive correlations in red and negative correlations in blue.

Supplementary Material 4: Figure S3. A) Principal component analysis (PCA) represents the separation of samples based on genotype and acclimation treatments. B) Heatmap of the top 500 differentially expressed genes, with hierarchical clustering revealing distinct expression modules primarily structured by acclimation treatments rather than genotype.

Supplementary Material 5: Figure S4. Differential gene expression (DEGs) patterns in CA vs DA in Col-4. A) Heatmaps showing up‑regulated and down‑regulated genes in Col‑4 in response to CA vs DA comparison as well as in BIL1 and GSK3. B) Heatmap of the top 500 DEGs (CA vs DA), highlighted the major transcriptional shifts between acclimation treatments.

Supplementary Material 6: Figure S5. DEGs patterns in CA vs RA in Col-4. A) Heatmaps showing up‑regulated and down‑regulated genes in Col‑4 in response to CA vs RA comparison as well as in BIL1 and GSK3. B) Heatmap of the top 500 DEGs (CA vs RA), highlighted the major transcriptional shifts between acclimation treatments.

Supplementary Material 7: Figure S6. DEGs patterns in CA vs DA in BIL1. A) Heatmaps showing up‑regulated and down‑regulated genes in BIL1 in response to CA vs DA comparison as well as in Col-4 and GSK3. B) Heatmap of the top 500 DEGs (CA vs DA), highlighted the major transcriptional shifts between acclimation treatments.

Supplementary Material 8: Figure S7. DEGs patterns in CA vs RA in BIL1. A) Heatmaps showing up‑regulated and down‑regulated genes in BIL1 in response to CA vs RA comparison as well as in Col-4 and GSK3. B) Heatmap of the top 500 DEGs (CA vs RA), highlighted the major transcriptional shifts between acclimation treatments.

Supplementary Material 9: Figure S8. DEGs patterns in CA vs DA in GSK3. A) Heatmaps showing up‑regulated and down‑regulated genes in GSK3 in response to CA vs DA comparison as well as in Col-4 and BIL1. B) Heatmap of the top 500 DEGs (CA vs DA), highlighted the major transcriptional shifts between acclimation treatments.

Supplementary Material 10: Figure S9: DEGs patterns in CA vs RA in GSK3. A) Heatmaps showing up‑regulated and down‑regulated genes in GSK3 in response to CA vs RA comparison as well as in Col-4 and BIL1. B) Heatmap of the top 500 DEGs (CA vs RA), highlighted the major transcriptional shifts between acclimation treatments.

Supplementary Material 11: Figure S10. DEGs showed overlapping and unique responses among the genotypes and treatments. A) Up-regulated genes across all genotypes. B) Down regulation across all genotypes.

Supplementary Material 12: Figure S11. DEGs showed overlapping and unique responses among specific comparison of genotype across treatment groups. A) Up-regulated and down regulated genes of Col-4 comparison. B) Up-regulated and down regulated genes of BIL1 comparison. C) Up-regulated and down regulated genes of GSK3 comparison.

Supplementary Material 13: Figure S12. Gene Ontology (GO) enrichment analysis of differentially expressed genes in Col-4 across acclimation transitions. Dot plots show significantly enriched GO terms for up-regulated and down-regulated genes in Biological Process (BP), Cellular Component (CC), and Molecular Function (MP) categories for CA vs DA, CA vs RA, comparisons. Dot size represents the number of genes associated with each GO term, while color intensity indicates enrichment significance expressed as −log₁₀(adjusted P-value).

Supplementary Material 14: Figure S13. GO enrichment analysis of differentially expressed genes in BIL1 across acclimation transitions. Dot plots show significantly enriched GO terms for up-regulated and down-regulated genes in BP, CC, and MP categories for CA vs DA, CA vs RA, comparisons. Dot size represents the number of genes associated with each GO term, while color intensity indicates enrichment significance expressed as −log₁₀(adjusted P-value).

Supplementary Material 15: Figure S14. GO enrichment analysis of differentially expressed genes in GSK3 across acclimation transitions. Dot plots show significantly enriched GO terms for up-regulated and down-regulated genes in BP, CC, and MP categories for CA vs DA, CA vs RA, comparisons. Dot size represents the number of genes associated with each GO term, while color intensity indicates enrichment significance expressed as −log₁₀(adjusted P-value).

Supplementary Material 16: Figure S15. Expression of canonical cold- and BR-signalling genes across genotypes and acclimation phases. Heatmap of mean FPKM values (annotated in each cell) for cold-signalling genes (ICE1/SCRM, CBF1–CBF3/DREB1B–A, COR15A), BR-signalling components (BZR1, BES1/BZR2, BIN2) and the two targeted kinase loci (BIL1, At2g30980; GSK3, At1g06390) in Col-4, BIL1 and GSK3 in response to CA, DA and RA. Cell colour represents the per-gene z-score (row-scaled across the nine conditions; red = high, blue = low).

Supplementary Material 17: Figure S16. Weighted gene co-expression network analysis (WGCNA) of transcriptomic responses across genotypes and acclimation treatments. (A) Hierarchical clustering dendrogram of genes based on topological overlap, with identified co-expression modules shown by distinct colors. (B) Soft-threshold power selection showing scale-free topology fit index and mean connectivity used to construct the signed network. (C) Topological overlap matrix (TOM) heatmap illustrating network connectivity and modular structure, where warmer colors indicate higher gene co-expression. (D) Module–condition relationship heatmap showing Pearson correlations between module eigengenes and genotype × treatment combinations (Col-4, BIL1, GSK3 under CA, DA, and RA). Color intensity reflects correlation strength, with red and blue indicating positive and negative associations, respectively, highlighting condition-specific regulatory modules.

Supplementary Material 18: Figure S17. Hub-gene network structure and functional enrichment of genotype-specific modules. A) Co-expression network of the purple module identified in BIL1 under CA vs RA. B) Co-expression network of the light-yellow module identified in GSK3 under CA vs RA. C) GO in response to BP of the BIL1 CA vs RA purple module (D) GO in response to BP of the GSK3 CA vs RA light-yellow module.

## Data Availability

The datasets used in this study are publicly available in the University of Agriculture in Krakow repository under DOI: ID10.15576/REPOURK/2026.1.8 . They can be accessed at https://repo.ur.krakow.pl/info/researchdata/URKb338a34e59b74d009bf5aab60276c9f4 , with direct download available via https://pliki.urk.edu.pl/index.php/s/aXbLnSryAZDCXsd. The rest of analysed data are present in this MS.
